# Supplementary material for: Facultatively intrabacterial localization of a planthopper endosymbiont as an adaptation to its vertical transmission
Source: mSystems. 2024 Jun 27;9(7):e00634-24. doi: 10.1128/msystems.00634-24 (PMC11264691; doi:10.1128/msystems.00634-24)
Supplement: Supplemental material — Supplemental figures and tables. [file msystems.00634-24-s0001.pdf]

Supplemental Material

Facultatively intra-bacterial localization of a planthopper endosymbiont as an adaptation to its vertical transmission

Anna Michalik[1*#], Diego C. Franco[1, 2*], Teresa Szklarzewicz[1], Adam Stroiński[3], Piotr Łukasik[2#]
* Contributed equally
# Corresponding Author

Corresponding Authors: Anna Michalik, Piotr Łukasik
Email: a.michalik@uj.edu.pl, p.lukasik@uj.edu.pl

Figures:
Fig. S1. Intra-*Sulcia* localization of *Acetobacteraceae*-TRYOCC symbionts.
Fig. S2. PROMER alignments of *Acetobacteraceae TRYOCC* genome against the genomes of various representatives of Acetobacteraceae family
Fig. S3. PROMER alignments of *Sulcia* and *Vidania* genomes from *T. occidentalis* (Tropiduchidae) against genomes from *Callodictya krueperi* (Dictyopharidae).
Fig. S4. FISH visualization of *Sulcia* cells in the bacteriome.
Fig. S5. Scatter plot based on 433 completed symbionts' genomes deposited in the NCBI database showing that *Vidania* of *T. occidentalis* has one of the smallest genome reported so far for a stand-alone bacterial genome.

Tables
Table S1. The comparison of metabolic pathways of *Asia bogorensis W19, Kirkpatrickella* and *Acetobacteraceae*-TRYOCC.
Table S2. The comparison of gene set between *Sulcia*-TRYOCC and *Sulcia* lineages from eight planthopper species.
Table S3. The comparison of gene set between *Vidania*-TRYOCC and *Vidania* lineages from eight planthopper species.
Table S4. Completeness values for *Sulcia, Vidania* and *Acetobacteraceae* symbionts of *T. occidentalis* and other *Sulcia* symbionts of Auchenorrhyncha.
Table S5. Sequences of symbiont-specific fluorochrome-labelled oligonucleotide probes.
Table S6. Accession numbers of *Acetobacteraceae* genomes used for phylogenomic analysis.

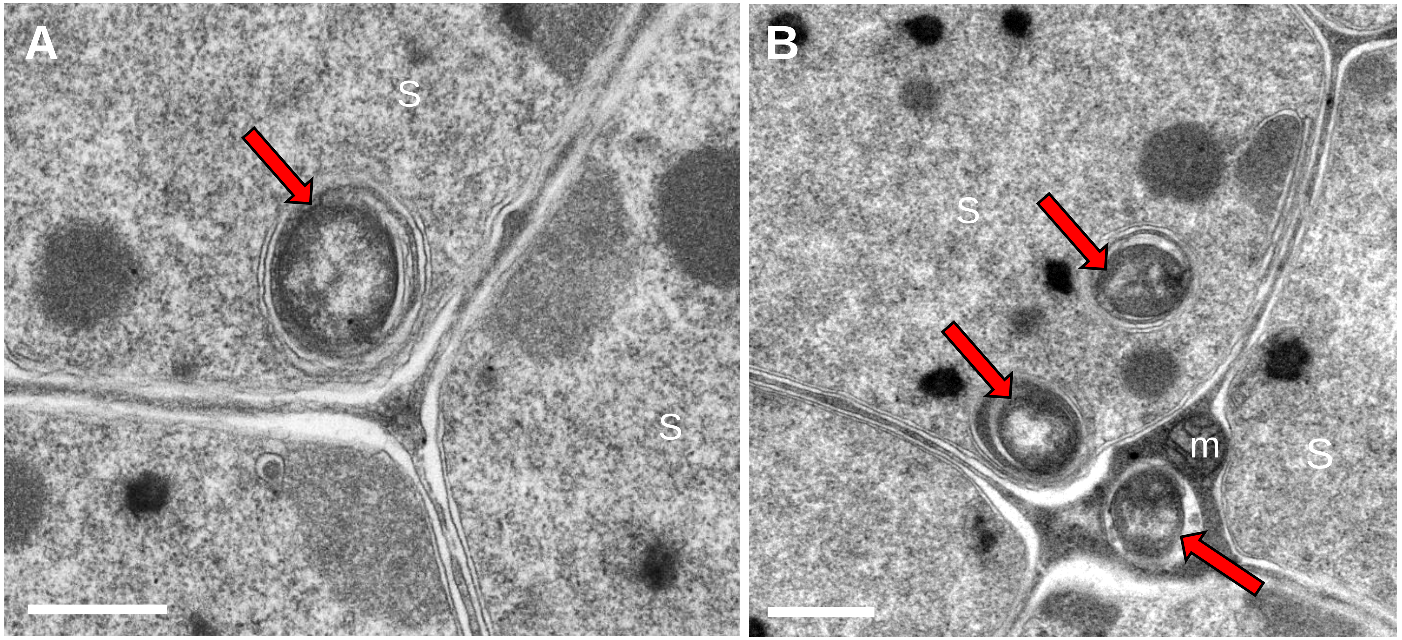

Fig. S1. Intra-*Sulcia* localization of *Acetobacteraceae*-TRYOCC symbionts. TEM, scale bar - 1 μm. m – mitochondrion, S – *Sulcia*, red arrow - *Acetobacteraceae*-TRYOCC symbiont.

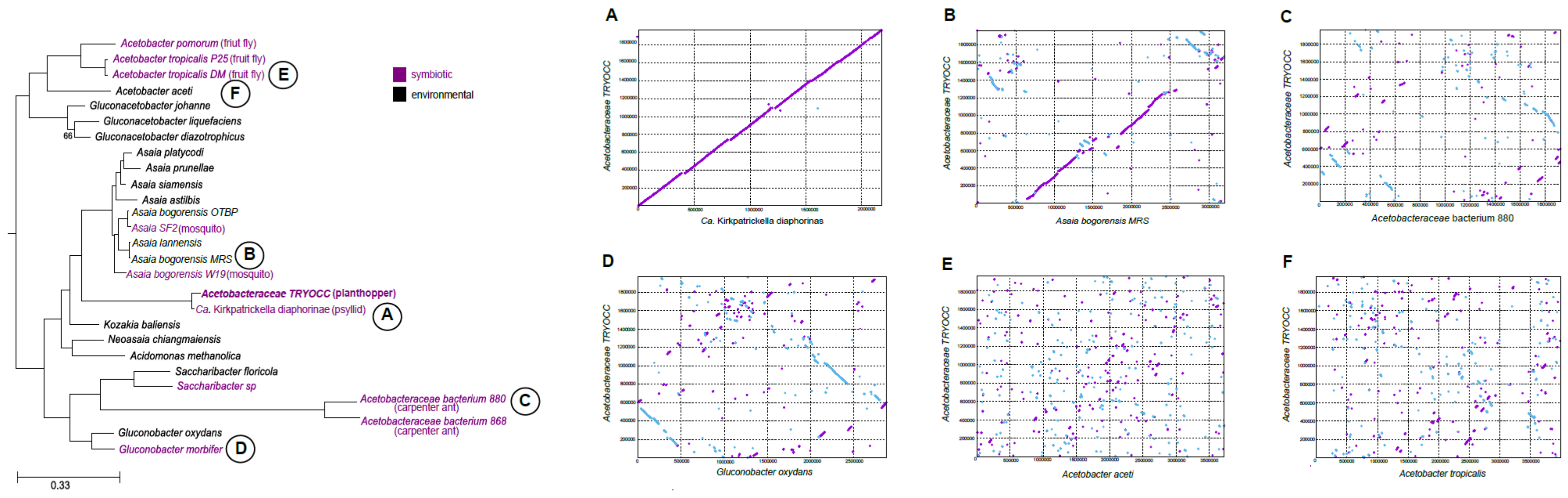

Fig. S2. PROMER alignments of *Acetobacteraceae TRYOCC* genome against the genomes of various representatives of Acetobacteraceae family: **A.** *Ca.* Kirkpatrickella diaphorinae B. *Asaia bogorensis MRS* **C.** *Acetobacteraceae bacterium 880* **D.** *Gluconobacter oxydans* **E.** *Acetobacter aceti* **F.** *Acetobacter tropicalis*. The bacterial phylogeny and panel A overlap with Figure 4.

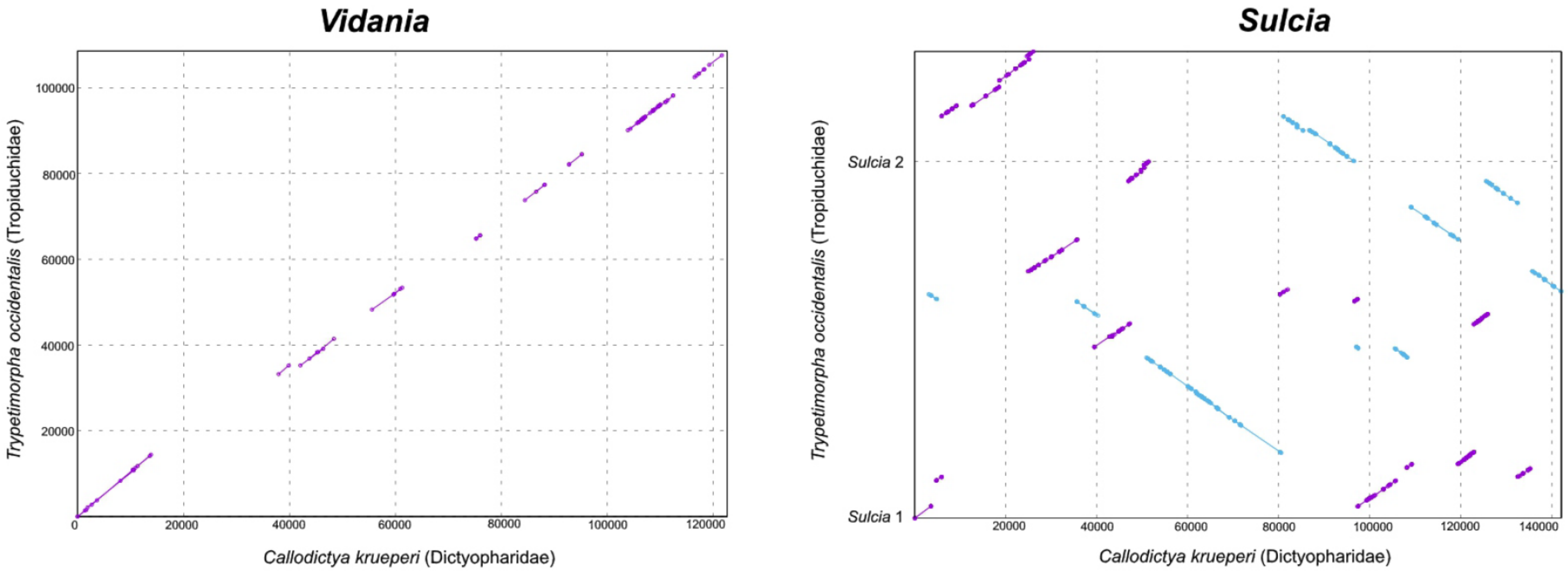

Fig. S3. PROMER alignments of *Sulcia* and *Vidania* genomes from *T. occidentalis* (Tropiduchidae) against genomes from *Callodictya krueperi* (Dictyopharidae).

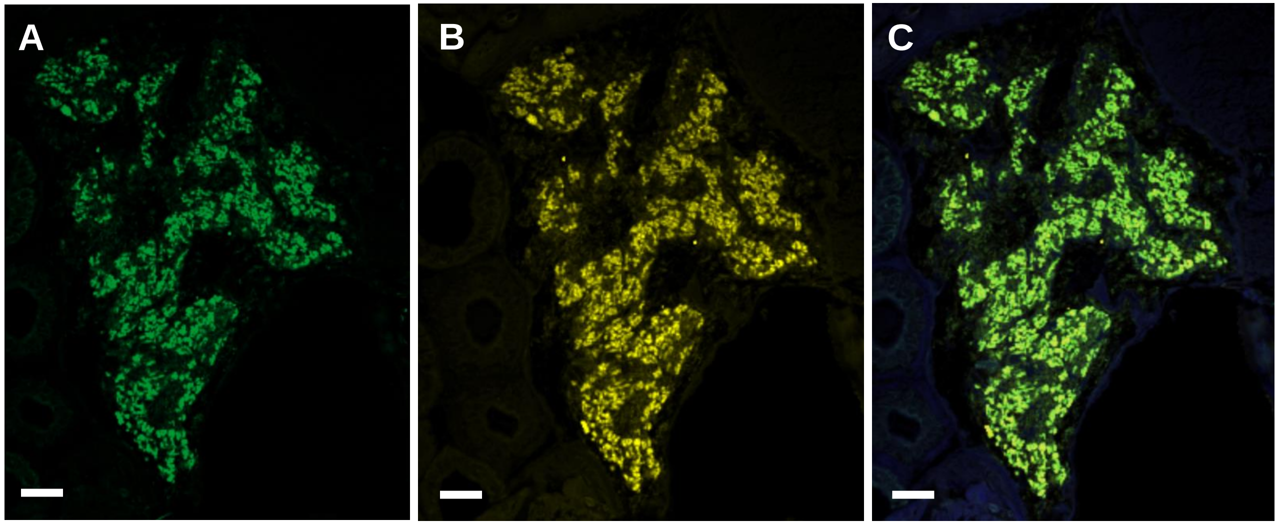

Fig. S4. FISH visualization of *Sulcia* cells in the bacteriome. CM, scale bar - 10 µm. A. Visualization of the *ilvB* gene localization in *Sulcia* cells (green). B. Visualization of *Sulcia* cells using a probe specific to 16S rDNA gene (yellow). C. Merged signals from *ilvB* and 16S rDNA-specific probes show the overlap between both signals and confirm that the large chromosome with *ilvB* gene is present in all *Sulcia* cells.

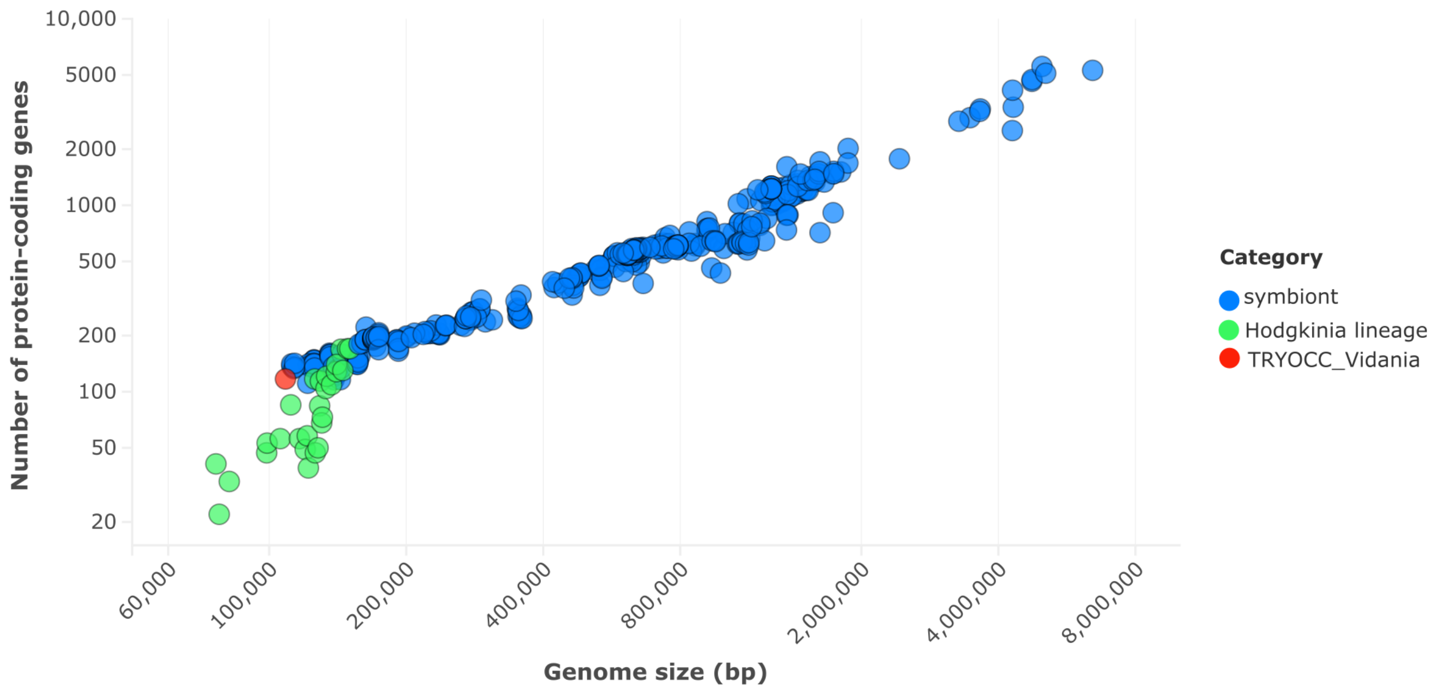

Fig. S5. Scatter plot based on 433 completed symbiont genomes deposited in the NCBI database showing that *Vidania* of *T. occidentalis* has one of the smallest genome reported so far for a stand-alone bacterial genome.

Table S1. The comparison of metabolic pathways of *Asia bogorensis W19*, *Kirkpatrickella* and *Acetobacteraceae TRYOCC*.

Asaia bogorensis_W19	***Ca.* Kirkpatrella diaphorinas**	***Acetobacteraceae TRYOCC***
**Carbohydrate metabolism**	**Carbohydrate metabolism**	**Carbohydrate metabolism**
Central carbohydrate metabolism	Central carbohydrate metabolism	Central carbohydrate metabolism
M00001 Glycolysis (Embden-Meyerhof pathway), glucose => pyruvate (10) (1 block missing 8/9)	M00001 Glycolysis (Embden-Meyerhof pathway), glucose => pyruvate (9) (2 blocks missing 7/9)	M00001 Glycolysis (Embden-Meyerhof pathway), glucose => pyruvate (9) (2 blocks missing 7/9)
M00002 Glycolysis, core module involving three-carbon compounds (6) (complete 5/5)	M00002 Glycolysis, core module involving three-carbon compounds (5) (1 block missing 4/5)	M00002 Glycolysis, core module involving three-carbon compounds (5) (1 block missing 4/5)
M00003 Gluconeogenesis, oxaloacetate => fructose-6P (8) (1 block missing 6/7)	M00003 Gluconeogenesis, oxaloacetate => fructose-6P (7) (2 blocks missing 5/7)	M00003 Gluconeogenesis, oxaloacetate => fructose-6P (7) (2 blocks missing 5/7)
M00307 Pyruvate oxidation, pyruvate => acetyl-CoA (5) (complete 1/1)	M00307 Pyruvate oxidation, pyruvate => acetyl-CoA (4) (complete 1/1)	M00307 Pyruvate oxidation, pyruvate => acetyl-CoA (4) (complete 1/1)
M00009 Citrate cycle (TCA cycle, Krebs cycle) (10) (2 blocks missing 6/8)	M00009 Citrate cycle (TCA cycle, Krebs cycle) (11) (2 blocks missing 6/8)	M00009 Citrate cycle (TCA cycle, Krebs cycle) (11) (2 blocks missing 6/8)
M00010 Citrate cycle, first carbon oxidation, oxaloacetate => 2-oxoglutarate (3) (complete 3/3)	M00010 Citrate cycle, first carbon oxidation, oxaloacetate => 2-oxoglutarate (3) (complete 3/3)	M00010 Citrate cycle, first carbon oxidation, oxaloacetate => 2-oxoglutarate (3) (complete 3/3)
M00011 Citrate cycle, second carbon oxidation, 2-oxoglutarate => oxaloacetate (7) (2 blocks missing 3/5)	M00011 Citrate cycle, second carbon oxidation, 2-oxoglutarate => oxaloacetate (8) (2 blocks missing 3/5)	M00011 Citrate cycle, second carbon oxidation, 2-oxoglutarate => oxaloacetate (8) (2 blocks missing 3/5)
M00004 Pentose phosphate pathway (Pentose phosphate cycle) (9) (complete 6/6)	M00004 Pentose phosphate pathway (Pentose phosphate cycle) (7) (complete 6/6)	M00004 Pentose phosphate pathway (Pentose phosphate cycle) (7) (complete 6/6)
M00006 Pentose phosphate pathway, oxidative phase, glucose 6P => ribulose 5P (4) (complete 2/2)	M00006 Pentose phosphate pathway, oxidative phase, glucose 6P => ribulose 5P (3) (complete 2/2)	M00006 Pentose phosphate pathway, oxidative phase, glucose 6P => ribulose 5P (3) (complete 2/2)
M00007 Pentose phosphate pathway, non-oxidative phase, fructose 6P => ribose 5P (5) (complete 4/4)	M00007 Pentose phosphate pathway, non-oxidative phase, fructose 6P => ribose 5P (4) (complete 4/4)	M00007 Pentose phosphate pathway, non-oxidative phase, fructose 6P => ribose 5P (4) (complete 4/4)
M00580 Pentose phosphate pathway, archaea, fructose 6P => ribose 5P (2) (1 block missing 1/2)	M00580 Pentose phosphate pathway, archaea, fructose 6P => ribose 5P (2) (1 block missing 1/2)	M00580 Pentose phosphate pathway, archaea, fructose 6P => ribose 5P (2) (1 block missing 1/2)
M00005 PRPP biosynthesis, ribose 5P => PRPP (1) (complete 1/1)	M00005 PRPP biosynthesis, ribose 5P => PRPP (1) (complete 1/1)	M00005 PRPP biosynthesis, ribose 5P => PRPP (1) (complete 1/1)
M00008 Entner-Doudoroff pathway, glucose-6P => glyceraldehyde-3P + pyruvate (5) (complete 4/4)	M00008 Entner-Doudoroff pathway, glucose-6P => glyceraldehyde-3P + pyruvate (2) (2 blocks missing 2/4)	M00008 Entner-Doudoroff pathway, glucose-6P => glyceraldehyde-3P + pyruvate (2) (2 blocks missing 2/4)
M00308 Semi-phosphorylative Entner-Doudoroff pathway, gluconate => glycerate-3P (3) (2 blocks missing 2/4)	M00308 Semi-phosphorylative Entner-Doudoroff pathway, gluconate => glycerate-3P (2) (incomplete 1/4)	M00308 Semi-phosphorylative Entner-Doudoroff pathway, gluconate => glycerate-3P (2) (incomplete 1/4)
Other carbohydrate metabolism	Other carbohydrate metabolism	Other carbohydrate metabolism
M00014 Glucuronate pathway (uronate pathway) (4) (incomplete 3/7)	M00014 Glucuronate pathway (uronate pathway) (2) (incomplete 2/7)	M00014 Glucuronate pathway (uronate pathway) (2) (incomplete 2/7)
M00631 D-Galacturonate degradation (bacteria), D-galacturonate => pyruvate + D-glyceraldehyde 3P (1) (incomplete 1/5)	**ABSENT**	**ABSENT**

M00061 D-Glucuronate degradation, D-glucuronate => pyruvate + D-glyceraldehyde 3P (1) (incomplete 1/5)	M00061 D-Glucuronate degradation, D-glucuronate => pyruvate + D-glyceraldehyde 3P (1) (incomplete 1/5)	M00061 D-Glucuronate degradation, D-glucuronate => pyruvate + D-glyceraldehyde 3P (1) (incomplete 1/5)
M00632 Galactose degradation, Leloir pathway, galactose => alpha-D-glucose-1P (2) (2 blocks missing 2/4)	M00632 Galactose degradation, Leloir pathway, galactose => alpha-D-glucose-1P (2) (2 blocks missing 2/4)	M00632 Galactose degradation, Leloir pathway, galactose => alpha-D-glucose-1P (1) (incomplete 1/4)
M00552 D-galactonate degradation, De Ley-Doudoroff pathway, D-galactonate => glycerate-3P (2) (incomplete 2/5)	M00552 D-galactonate degradation, De Ley-Doudoroff pathway, D-galactonate => glycerate-3P (2) (incomplete 2/5)	M00552 D-galactonate degradation, De Ley-Doudoroff pathway, D-galactonate => glycerate-3P (2) (incomplete 2/5)
M00129 Ascorbate biosynthesis, animals, glucose-1P => ascorbate (3) (incomplete 3/7)	M00129 Ascorbate biosynthesis, animals, glucose-1P => ascorbate (3) (incomplete 3/7)	M00129 Ascorbate biosynthesis, animals, glucose-1P => ascorbate (2) (incomplete 2/7)
M00854 Glycogen biosynthesis, glucose-1P => glycogen/starch (3) (1 block missing 1/2)	ABSENT	ABSENT
M00855 Glycogen degradation, glycogen => glucose-6P (1) (2 blocks missing 1/3)	M00855 Glycogen degradation, glycogen => glucose-6P (1) (2 blocks missing 1/3)	M00855 Glycogen degradation, glycogen => glucose-6P (1) (2 blocks missing 1/3)
M00565 Trehalose biosynthesis, D-glucose 1P => trehalose (5) (1 block missing 5/6)	ABSENT	ABSENT
M00549 Nucleotide sugar biosynthesis, glucose => UDP-glucose (3) (complete 3/3)	M00549 Nucleotide sugar biosynthesis, glucose => UDP-glucose (3) (complete 3/3)	M00549 Nucleotide sugar biosynthesis, glucose => UDP-glucose (3) (complete 3/3)
M00892 UDP-N-acetyl-D-glucosamine biosynthesis, eukaryotes, glucose => UDP-GlcNAc (2) (incomplete 2/6)	M00892 UDP-N-acetyl-D-glucosamine biosynthesis, eukaryotes, glucose => UDP-GlcNAc (2) (incomplete 2/6)	M00892 UDP-N-acetyl-D-glucosamine biosynthesis, eukaryotes, glucose => UDP-GlcNAc (2) (incomplete 2/6)
M00909 UDP-N-acetyl-D-glucosamine biosynthesis, prokaryotes, glucose => UDP-GlcNAc (5) (complete 5/5)	M00909 UDP-N-acetyl-D-glucosamine biosynthesis, prokaryotes, glucose => UDP-GlcNAc (5) (complete 5/5)	M00909 UDP-N-acetyl-D-glucosamine biosynthesis, prokaryotes, glucose => UDP-GlcNAc (5) (complete 5/5)
M00012 Glyoxylate cycle (2) (incomplete 2/5)	M00012 Glyoxylate cycle (2) (incomplete 2/5)	M00012 Glyoxylate cycle (2) (incomplete 2/5)
M00740 Methylaspartate cycle (2) (incomplete 2/11)	M00740 Methylaspartate cycle (2) (incomplete 2/11)	M00740 Methylaspartate cycle (2) (incomplete 2/11)
M00532 Photorespiration (6) (incomplete 3/10)	M00532 Photorespiration (7) (incomplete 4/10)	M00532 Photorespiration (5) (incomplete 2/10)
M00013 Malonate semialdehyde pathway, propanoyl-CoA => acetyl-CoA (1) (incomplete 1/5)	ABSENT	ABSENT
M00131 Inositol phosphate metabolism, Ins(1,3,4,5)P4 => Ins(1,3,4)P3 => myo-inositol (1) (incomplete 1/4)	M00131 Inositol phosphate metabolism, Ins(1,3,4,5)P4 => Ins(1,3,4)P3 => myo-inositol (1) (incomplete 1/4)	M00131 Inositol phosphate metabolism, Ins(1,3,4,5)P4 => Ins(1,3,4)P3 => myo-inositol (1) (incomplete 1/4)
**Energy metabolism**	**Energy metabolism**	**Energy metabolism**
Carbon fixation	Carbon fixation	Carbon fixation
M00165 Reductive pentose phosphate cycle (Calvin cycle) (8) (2 blocks missing 9/11)	M00165 Reductive pentose phosphate cycle (Calvin cycle) (7) (incomplete 8/11)	M00165 Reductive pentose phosphate cycle (Calvin cycle) (7) (incomplete 8/11)
M00168 CAM (Crassulacean acid metabolism), dark (1) (1 block missing 1/2)	M00168 CAM (Crassulacean acid metabolism), dark (1) (1 block missing 1/2)	M00168 CAM (Crassulacean acid metabolism), dark (1) (1 block missing 1/2)
M00172 C4-dicarboxylic acid cycle, NADP - malic enzyme type (1) (incomplete 1/4)	M00172 C4-dicarboxylic acid cycle, NADP - malic enzyme type (1) (incomplete 1/4)	M00172 C4-dicarboxylic acid cycle, NADP - malic enzyme type (1) (incomplete 1/4)
M00171 C4-dicarboxylic acid cycle, NAD - malic enzyme type (1) (incomplete 1/7)	M00171 C4-dicarboxylic acid cycle, NAD - malic enzyme type (1) (incomplete 1/7)	M00171 C4-dicarboxylic acid cycle, NAD - malic enzyme type (1) (incomplete 1/7)
M00170 C4-dicarboxylic acid cycle, phosphoenolpyruvate carboxykinase type (1) (incomplete 1/4)	M00170 C4-dicarboxylic acid cycle, phosphoenolpyruvate carboxykinase type (1) (incomplete 1/4)	M00170 C4-dicarboxylic acid cycle, phosphoenolpyruvate carboxykinase type (1) (incomplete 1/4)
M00173 Reductive citrate cycle (Arnon-Buchanan cycle) (7) (incomplete 3/10)	M00173 Reductive citrate cycle (Arnon-Buchanan cycle) (7) (incomplete 3/10)	M00173 Reductive citrate cycle (Arnon-Buchanan cycle) (7) (incomplete 3/10)

M00376 3-Hydroxypropionate bi-cycle (7) (incomplete 2/13)	M00376 3-Hydroxypropionate bi-cycle (8) (incomplete 3/13)	M00376 3-Hydroxypropionate bi-cycle (8) (incomplete 3/13)
M00374 Dicarboxylate-hydroxybutyrate cycle (3) (incomplete 1/13)	M00374 Dicarboxylate-hydroxybutyrate cycle (4) (incomplete 2/13)	M00374 Dicarboxylate-hydroxybutyrate cycle (4) (incomplete 2/13)
M00377 Reductive acetyl-CoA pathway (Wood-Ljungdahl pathway) (1) (incomplete 1/7)	M00377 Reductive acetyl-CoA pathway (Wood-Ljungdahl pathway) (1) (incomplete 1/7)	M00377 Reductive acetyl-CoA pathway (Wood-Ljungdahl pathway) (1) (incomplete 1/7)
M00579 Phosphate acetyltransferase-acetate kinase pathway, acetyl-CoA => acetate (2) (complete 2/2)	**ABSENT**	**ABSENT**
Methane metabolism	Methane metabolism	Methane metabolism
M00357 Methanogenesis, acetate => methane (2) (incomplete 1/5)	M00357 Methanogenesis, acetate => methane (1) (incomplete 1/5)	**ABSENT**
M00346 Formaldehyde assimilation, serine pathway (3) (incomplete 3/9)	M00346 Formaldehyde assimilation, serine pathway (3) (incomplete 3/9)	M00346 Formaldehyde assimilation, serine pathway (3) (incomplete 3/9)
M00345 Formaldehyde assimilation, ribulose monophosphate pathway (2) (2 blocks missing 1/3)	M00345 Formaldehyde assimilation, ribulose monophosphate pathway (2) (2 blocks missing 1/3)	M00345 Formaldehyde assimilation, ribulose monophosphate pathway (2) (2 blocks missing 1/3)
M00344 Formaldehyde assimilation, xylulose monophosphate pathway (2) (2 blocks missing 2/4)	M00344 Formaldehyde assimilation, xylulose monophosphate pathway (1) (incomplete 1/4)	M00344 Formaldehyde assimilation, xylulose monophosphate pathway (1) (incomplete 1/4)
Sulfur metabolism	Sulfur metabolism	**ABSENT**
M00176 Assimilatory sulfate reduction, sulfate => H2S (4) (1 block missing 1/2)	M00176 Assimilatory sulfate reduction, sulfate => H2S (4) (1 block missing 1/2)	
ATP synthesis	ATP synthesis	ATP synthesis
M00144 NADH:quinone oxidoreductase, prokaryotes (13) (complete 1/1)	M00144 NADH:quinone oxidoreductase, prokaryotes (13) (complete 1/1)	M00144 NADH:quinone oxidoreductase, prokaryotes (13) (complete 1/1)
	M00149 Succinate dehydrogenase, prokaryotes (4) (complete 1/1)	M00149 Succinate dehydrogenase, prokaryotes (4) (complete 1/1)
M00417 Cytochrome o ubiquinol oxidase (4) (complete 1/1)	M00417 Cytochrome o ubiquinol oxidase (4) (complete 1/1)	M00417 Cytochrome o ubiquinol oxidase (4) (complete 1/1)
**Lipid metabolism**	**Lipid metabolism**	**Lipid metabolism**
Fatty acid metabolism	Fatty acid metabolism	Fatty acid metabolism
M00082 Fatty acid biosynthesis, initiation (6) (complete 2/2)	M00082 Fatty acid biosynthesis, initiation (6) (complete 2/2)	M00082 Fatty acid biosynthesis, initiation (6) (complete 2/2)
M00083 Fatty acid biosynthesis, elongation (5) (complete 1/1)	M00083 Fatty acid biosynthesis, elongation (5) (complete 1/1)	M00083 Fatty acid biosynthesis, elongation (5) (complete 1/1)
M00873 Fatty acid biosynthesis in mitochondria, animals (2) (incomplete 1/6)	M00873 Fatty acid biosynthesis in mitochondria, animals (2) (incomplete 1/6)	M00873 Fatty acid biosynthesis in mitochondria, animals (2) (incomplete 1/6)
M00874 Fatty acid biosynthesis in mitochondria, fungi (3) (incomplete 2/6)	M00874 Fatty acid biosynthesis in mitochondria, fungi (3) (incomplete 2/6)	M00874 Fatty acid biosynthesis in mitochondria, fungi (3) (incomplete 2/6)
Lipid metabolism	Lipid metabolism	Lipid metabolism
M00089 Triacylglycerol biosynthesis (1) (incomplete 1/4)	M00089 Triacylglycerol biosynthesis (1) (incomplete 1/4)	M00089 Triacylglycerol biosynthesis (1) (incomplete 1/4)
M00091 Phosphatidylcholine (PC) biosynthesis, PE => PC (1) (complete 1/1)	M00091 Phosphatidylcholine (PC) biosynthesis, PE => PC (1) (complete 1/1)	M00091 Phosphatidylcholine (PC) biosynthesis, PE => PC (1) (complete 1/1)

M00093 Phosphatidylethanolamine (PE) biosynthesis, PA => PS => PE (3) (complete 3/3)	M00093 Phosphatidylethanolamine (PE) biosynthesis, PA => PS => PE (3) (complete 3/3)	M00093 Phosphatidylethanolamine (PE) biosynthesis, PA => PS => PE (3) (complete 3/3)
M00066 Lactosylceramide biosynthesis (1) (1 block missing 1/2)	M00066 Lactosylceramide biosynthesis (1) (1 block missing 1/2)	M00066 Lactosylceramide biosynthesis (1) (1 block missing 1/2)
**Nucleotide metabolism**	**Nucleotide metabolism**	**Nucleotide metabolism**
Purine metabolism	Purine metabolism	Purine metabolism
M00048 De novo purine biosynthesis, PRPP + glutamine => IMP (13) (complete 8/8)	M00048 De novo purine biosynthesis, PRPP + glutamine => IMP (12) (complete 8/8)	M00048 De novo purine biosynthesis, PRPP + glutamine => IMP (12) (complete 8/8)
M00049 Adenine ribonucleotide biosynthesis, IMP => ADP,ATP (4) (complete 4/4)	M00049 Adenine ribonucleotide biosynthesis, IMP => ADP,ATP (3) (1 block missing 3/4) complete - missing gene manually found in the genome	M00049 Adenine ribonucleotide biosynthesis, IMP => ADP,ATP (4) (complete 4/4)
M00050 Guanine ribonucleotide biosynthesis, IMP => GDP,GTP (4) (complete 4/4)	M00050 Guanine ribonucleotide biosynthesis, IMP => GDP,GTP (3) (1 block missing 3/4) complete - missing gene manually found in the genome	M00050 Guanine ribonucleotide biosynthesis, IMP => GDP,GTP (4) (complete 4/4)
M00053 Deoxyribonucleotide biosynthesis, ADP/GDP/CDP/UDP => dATP/dGTP/dCTP/dUTP (3) (complete 2/2)	M00053 Deoxyribonucleotide biosynthesis, ADP/GDP/CDP/UDP => dATP/dGTP/dCTP/dUTP (2) (1 block missing 1/2) complete - missing gene manually found in the genome	M00053 Deoxyribonucleotide biosynthesis, ADP/GDP/CDP/UDP => dATP/dGTP/dCTP/dUTP (3) (complete 2/2)
M00958 Adenine ribonucleotide degradation, AMP => Urate (5) (complete 3/3)	M00958 Adenine ribonucleotide degradation, AMP => Urate (2) (1 block missing 2/3)	ABSENT
M00959 Guanine ribonucleotide degradation, GMP => Urate (5) (complete 4/4)	M00959 Guanine ribonucleotide degradation, GMP => Urate (2) (2 blocks missing 2/4)	M00959 Guanine ribonucleotide degradation, GMP => Urate (1) (incomplete 1/4)
M00546 Purine degradation, xanthine => urea (6) (1 block missing 4/5)	M00546 Purine degradation, xanthine => urea (1) (incomplete 1/5)	M00546 Purine degradation, xanthine => urea (1) (incomplete 1/5)
Pyrimidine metabolism	Pyrimidine metabolism	Pyrimidine metabolism
M00051 De novo pyrimidine biosynthesis, glutamine (+ PRPP) => UMP (7) (1 block missing 2/3)	M00051 De novo pyrimidine biosynthesis, glutamine (+ PRPP) => UMP (7) (1 block missing 2/3)	M00051 De novo pyrimidine biosynthesis, glutamine (+ PRPP) => UMP (7) (1 block missing 2/3)
M00052 Pyrimidine ribonucleotide biosynthesis, UMP => UDP/UTP,CDP/CTP (3) (complete 3/3)	M00052 Pyrimidine ribonucleotide biosynthesis, UMP => UDP/UTP,CDP/CTP (2) (1 block missing 2/3) complete - missing gene manually found in the genome	M00052 Pyrimidine ribonucleotide biosynthesis, UMP => UDP/UTP,CDP/CTP (3) (complete 3/3)
M00938 Pyrimidine deoxyribonucleotide biosynthesis, UDP => dTTP (6) (complete 5/5)	M00938 Pyrimidine deoxyribonucleotide biosynthesis, UDP => dTTP (5) (1 block missing 4/5) complete - missing gene manually found in the genome	M00938 Pyrimidine deoxyribonucleotide biosynthesis, UDP => dTTP (6) (complete 5/5)
M00046 Pyrimidine degradation, uracil => beta-alanine, thymine => 3-aminoisobutanoate (4) (complete 3/3)	ABSENT	ABSENT
M00939 Pyrimidine degradation, uracil => 3-hydroxypropanoate (3) (incomplete 1/5)	M00939 Pyrimidine degradation, uracil => 3-hydroxypropanoate (2) (incomplete 1/5)	M00939 Pyrimidine degradation, uracil => 3-hydroxypropanoate (2) (incomplete 1/5)
**Amino acid metabolism**	**Amino acid metabolism**	**Amino acid metabolism**
Serine and threonine metabolism	Serine and threonine metabolism	Serine and threonine metabolism
M00020 Serine biosynthesis, glycerate-3P => serine (3) (complete 3/3)	M00020 Serine biosynthesis, glycerate-3P => serine (3) (complete 3/3)	M00020 Serine biosynthesis, glycerate-3P => serine (3) (complete 3/3)
M00018 Threonine biosynthesis, aspartate => homoserine => threonine (5) (complete 5/5)	M00018 Threonine biosynthesis, aspartate => homoserine => threonine (5) (complete 5/5)	M00018 Threonine biosynthesis, aspartate => homoserine => threonine (5) (complete 5/5)

M00621 Glycine cleavage system (3) (complete 3/3)	M00621 Glycine cleavage system (3) (complete 3/3)	M00621 Glycine cleavage system (3) (complete 3/3)
M00033 Ectoine biosynthesis, aspartate => ectoine (2) (incomplete 2/5)	M00033 Ectoine biosynthesis, aspartate => ectoine (2) (incomplete 2/5)	M00033 Ectoine biosynthesis, aspartate => ectoine (2) (incomplete 2/5)
Cysteine and methionine metabolism	Cysteine and methionine metabolism	Cysteine and methionine metabolism
M00021 Cysteine biosynthesis, serine => cysteine (2) (complete 2/2)	M00021 Cysteine biosynthesis, serine => cysteine (2) (complete 2/2)	M00021 Cysteine biosynthesis, serine => cysteine (2) (complete 2/2)
M00609 Cysteine biosynthesis, methionine => cysteine (1) (incomplete 1/6)	M00609 Cysteine biosynthesis, methionine => cysteine (1) (incomplete 1/6)	M00609 Cysteine biosynthesis, methionine => cysteine (1) (incomplete 1/6)
M00017 Methionine biosynthesis, aspartate => homoserine => methionine (7) (1 block missing 6/7)	M00017 Methionine biosynthesis, aspartate => homoserine => methionine (7) (1 block missing 6/7)	M00017 Methionine biosynthesis, aspartate => homoserine => methionine (5) (2 blocks missing 5/7)
M00034 Methionine salvage pathway (10) (1 block missing 7/8)	M00034 Methionine salvage pathway (5) (incomplete 4/8)	M00034 Methionine salvage pathway (4) (incomplete 4/8)
M00035 Methionine degradation (3) (1 block missing 3/4)	M00035 Methionine degradation (2) (2 blocks missing 2/4)	M00035 Methionine degradation (2) (2 blocks missing 2/4)
M00368 Ethylene biosynthesis, methionine => ethylene (1) (2 blocks missing 1/3)	M00368 Ethylene biosynthesis, methionine => ethylene (1) (2 blocks missing 1/3)	M00368 Ethylene biosynthesis, methionine => ethylene (1) (2 blocks missing 1/3)
Branched-chain amino acid metabolism	Branched-chain amino acid metabolism	Branched-chain amino acid metabolism
M00019 Valine/isoleucine biosynthesis, pyruvate => valine / 2-oxobutanoate => isoleucine (5) (complete 4/4)	M00019 Valine/isoleucine biosynthesis, pyruvate => valine / 2-oxobutanoate => isoleucine (5) (complete 4/4)	M00019 Valine/isoleucine biosynthesis, pyruvate => valine / 2-oxobutanoate => isoleucine (5) (complete 4/4)
M00535 Isoleucine biosynthesis, pyruvate => 2-oxobutanoate (3) (1 block missing 2/3)	M00535 Isoleucine biosynthesis, pyruvate => 2-oxobutanoate (3) (1 block missing 2/3)	M00535 Isoleucine biosynthesis, pyruvate => 2-oxobutanoate (2) (2 blocks missing 1/3)
M00570 Isoleucine biosynthesis, threonine => 2-oxobutanoate => isoleucine (6) (complete 5/5)	M00570 Isoleucine biosynthesis, threonine => 2-oxobutanoate => isoleucine (6) (complete 5/5)	M00570 Isoleucine biosynthesis, threonine => 2-oxobutanoate => isoleucine (5) (1 block missing 4/5)
M00432 Leucine biosynthesis, 2-oxoisovalerate => 2-oxoisocaproate (4) (complete 3/3)	M00432 Leucine biosynthesis, 2-oxoisovalerate => 2-oxoisocaproate (4) (complete 3/3)	M00432 Leucine biosynthesis, 2-oxoisovalerate => 2-oxoisocaproate (3) (1 block missing 2/3)
M00036 Leucine degradation, leucine => acetoacetate + acetyl-CoA (2) (incomplete 1/6)	M00036 Leucine degradation, leucine => acetoacetate + acetyl-CoA (2) (incomplete 1/6)	M00036 Leucine degradation, leucine => acetoacetate + acetyl-CoA (2) (incomplete 1/6)
Lysine metabolism	Lysine metabolism	Lysine metabolism
M00016 Lysine biosynthesis, succinyl-DAP pathway, aspartate => lysine (10) (complete 9/9)	M00016 Lysine biosynthesis, succinyl-DAP pathway, aspartate => lysine (9) (complete 9/9)	M00016 Lysine biosynthesis, succinyl-DAP pathway, aspartate => lysine (9) (complete 9/9)
M00525 Lysine biosynthesis, acetyl-DAP pathway, aspartate => lysine (6) (incomplete 6/9)	M00525 Lysine biosynthesis, acetyl-DAP pathway, aspartate => lysine (6) (incomplete 6/9)	M00525 Lysine biosynthesis, acetyl-DAP pathway, aspartate => lysine (6) (incomplete 6/9)
M00526 Lysine biosynthesis, DAP dehydrogenase pathway, aspartate => lysine (5) (1 block missing 5/6)	M00526 Lysine biosynthesis, DAP dehydrogenase pathway, aspartate => lysine (5) (1 block missing 5/6)	M00526 Lysine biosynthesis, DAP dehydrogenase pathway, aspartate => lysine (5) (1 block missing 5/6)
M00527 Lysine biosynthesis, DAP aminotransferase pathway, aspartate => lysine (6) (1 block missing 6/7)	M00527 Lysine biosynthesis, DAP aminotransferase pathway, aspartate => lysine (6) (1 block missing 6/7)	M00527 Lysine biosynthesis, DAP aminotransferase pathway, aspartate => lysine (6) (1 block missing 6/7)
M00956 Lysine degradation, bacteria, L-lysine => succinate (1) (incomplete 1/7)	**ABSENT**	**ABSENT**
M00957 Lysine degradation, bacteria, L-lysine => glutarate => succinate/acetyl-CoA (1) (incomplete 1/5)	**ABSENT**	**ABSENT**
M00960 Lysine degradation, bacteria, L-lysine => D-lysine => succinate (2) (incomplete 2/9)	M00960 Lysine degradation, bacteria, L-lysine => D-lysine => succinate (1) (incomplete 1/9)	M00960 Lysine degradation, bacteria, L-lysine => D-lysine => succinate (1) (incomplete 1/9)
Arginine and proline metabolism	Arginine and proline metabolism	Arginine and proline metabolism

M00028 Ornithine biosynthesis, glutamate => ornithine (5) (complete 4/4)	M00028 Ornithine biosynthesis, glutamate => ornithine (4) (complete 4/4)	M00028 Ornithine biosynthesis, glutamate => ornithine (4) (complete 4/4)
M00844 Arginine biosynthesis, ornithine => arginine (3) (complete 3/3)	M00844 Arginine biosynthesis, ornithine => arginine (3) (complete 3/3)	M00844 Arginine biosynthesis, ornithine => arginine (3) (complete 3/3)
M00845 Arginine biosynthesis, glutamate => acetylcitrulline => arginine (5) (2 blocks missing 5/7)	M00845 Arginine biosynthesis, glutamate => acetylcitrulline => arginine (4) (incomplete 4/7)	M00845 Arginine biosynthesis, glutamate => acetylcitrulline => arginine (4) (incomplete 4/7)
M00029 Urea cycle (4) (1 block missing 4/5)	M00029 Urea cycle (3) (2 blocks missing 3/5)	M00029 Urea cycle (3) (2 blocks missing 3/5)
M00015 Proline biosynthesis, glutamate => proline (3) (complete 2/2)	M00015 Proline biosynthesis, glutamate => proline (3) (complete 2/2)	M00015 Proline biosynthesis, glutamate => proline (3) (complete 2/2)
M00970 Proline degradation, proline => glutamate (1) (complete 1/1)	M00970 Proline degradation, proline => glutamate (1) (complete 1/1)	**ABSENT**
M00972 Proline metabolism (1) (2 blocks missing 1/3)	M00972 Proline metabolism (1) (2 blocks missing 1/3)	M00972 Proline metabolism (1) (2 blocks missing 1/3)
Polyamine biosynthesis	Polyamine biosynthesis	Polyamine biosynthesis
M00133 Polyamine biosynthesis, arginine => agmatine => putrescine => spermidine (2) (2 blocks missing 2/4)	M00133 Polyamine biosynthesis, arginine => agmatine => putrescine => spermidine (2) (2 blocks missing 2/4)	M00133 Polyamine biosynthesis, arginine => agmatine => putrescine => spermidine (2) (2 blocks missing 2/4)
M00134 Polyamine biosynthesis, arginine => ornithine => putrescine (2) (complete 2/2)	M00134 Polyamine biosynthesis, arginine => ornithine => putrescine (1) (1 block missing 1/2)	M00134 Polyamine biosynthesis, arginine => ornithine => putrescine (1) (1 block missing 1/2)
M00136 GABA biosynthesis, prokaryotes, putrescine => GABA (1) (incomplete 1/4)		
Histidine metabolism	Histidine metabolism	Histidine metabolism
M00026 Histidine biosynthesis, PRPP => histidine (9) (1 block missing 5/6)	M00026 Histidine biosynthesis, PRPP => histidine (11) (complete 6/6)	M00026 Histidine biosynthesis, PRPP => histidine (6) (incomplete 2/6)
M00045 Histidine degradation, histidine => N-formiminoglutamate => glutamate (5) (complete 4/4)	**ABSENT**	**ABSENT**
Aromatic amino acid metabolism	Aromatic amino acid metabolism	Aromatic amino acid metabolism
M00022 Shikimate pathway, phosphoenolpyruvate + erythrose-4P => chorismate (6) (complete 4/4)	M00022 Shikimate pathway, phosphoenolpyruvate + erythrose-4P => chorismate (6) (complete 4/4)	M00022 Shikimate pathway, phosphoenolpyruvate + erythrose-4P => chorismate (6) (complete 4/4)
M00023 Tryptophan biosynthesis, chorismate => tryptophan (7) (complete 3/3)	M00023 Tryptophan biosynthesis, chorismate => tryptophan (7) (complete 3/3)	M00023 Tryptophan biosynthesis, chorismate => tryptophan (7) (complete 3/3)
M00024 Phenylalanine biosynthesis, chorismate => phenylpyruvate => phenylalanine (2) (1 block missing 1/2)	M00024 Phenylalanine biosynthesis, chorismate => phenylpyruvate => phenylalanine (2) (1 block missing 1/2)	M00024 Phenylalanine biosynthesis, chorismate => phenylpyruvate => phenylalanine (2) (1 block missing 1/2)
M00040 Tyrosine biosynthesis, chorismate => arogenate => tyrosine (2) (1 block missing 2/3)	M00040 Tyrosine biosynthesis, chorismate => arogenate => tyrosine (2) (1 block missing 2/3)	M00040 Tyrosine biosynthesis, chorismate => arogenate => tyrosine (2) (1 block missing 2/3)
M00042 Catecholamine biosynthesis, tyrosine => dopamine => noradrenaline => adrenaline (1) (incomplete 1/4)	**ABSENT**	**ABSENT**
M00037 Melatonin biosynthesis, animals, tryptophan => serotonin => melatonin (1) (incomplete 1/4)	M00037 Melatonin biosynthesis, animals, tryptophan => serotonin => melatonin (1) (incomplete 1/4)	**ABSENT**
M00936 Melatonin biosynthesis, plants, tryptophan => serotonin => melatonin (1) (incomplete 1/4)	**ABSENT**	**ABSENT**
Other amino acid metabolism	Other amino acid metabolism	Other amino acid metabolism
M00027 GABA (gamma-Aminobutyrate) shunt (1) (2 blocks missing 1/3)	**ABSENT**	**ABSENT**

M00118 Glutathione biosynthesis, glutamate => glutathione (2) (complete 2/2)	M00118 Glutathione biosynthesis, glutamate => glutathione (2) (complete 2/2)	M00118 Glutathione biosynthesis, glutamate => glutathione (2) (complete 2/2)
**Glycan metabolism**	**Glycan metabolism**	**Glycan metabolism**
Glycosaminoglycan metabolism	**ABSENT**	**ABSENT**
M00079 Keratan sulfate degradation (1) (incomplete 1/4)		
Lipopolysaccharide metabolism	Lipopolysaccharide metabolism	Lipopolysaccharide metabolism
M00060 KDO2-lipid A biosynthesis, Raetz pathway, LpxL-LpxM type (8) (1 block missing 8/9)	M00060 KDO2-lipid A biosynthesis, Raetz pathway, LpxL-LpxM type (8) (1 block missing 8/9)	M00060 KDO2-lipid A biosynthesis, Raetz pathway, LpxL-LpxM type (8) (1 block missing 8/9)
M00866 KDO2-lipid A biosynthesis, Raetz pathway, non-LpxL-LpxM type (8) (1 block missing 8/9)	M00866 KDO2-lipid A biosynthesis, Raetz pathway, non-LpxL-LpxM type (8) (1 block missing 8/9)	M00866 KDO2-lipid A biosynthesis, Raetz pathway, non-LpxL-LpxM type (8) (1 block missing 8/9)
M00867 KDO2-lipid A modification pathway (1) (incomplete 1/5)	M00867 KDO2-lipid A modification pathway (1) (incomplete 1/5)	M00867 KDO2-lipid A modification pathway (1) (incomplete 1/5)
M00063 CMP-KDO biosynthesis (3) (1 block missing 3/4)	M00063 CMP-KDO biosynthesis (3) (1 block missing 3/4)	M00063 CMP-KDO biosynthesis (3) (1 block missing 3/4)
M00064 ADP-L-glycero-D-manno-heptose biosynthesis (2) (2 blocks missing 3/5)	M00064 ADP-L-glycero-D-manno-heptose biosynthesis (3) (1 block missing 4/5)	M00064 ADP-L-glycero-D-manno-heptose biosynthesis (3) (1 block missing 4/5)
**Metabolism of cofactors and vitamins**	**Metabolism of cofactors and vitamins**	**Metabolism of cofactors and vitamins**
Cofactor and vitamin metabolism	Cofactor and vitamin metabolism	Cofactor and vitamin metabolism
M00127 Thiamine biosynthesis, prokaryotes, AIR (+ DXP/tyrosine) => TMP/TPP (6) (incomplete 4/7)	M00127 Thiamine biosynthesis, prokaryotes, AIR (+ DXP/tyrosine) => TMP/TPP (6) (incomplete 4/7)	M00127 Thiamine biosynthesis, prokaryotes, AIR (+ DXP/tyrosine) => TMP/TPP (6) (incomplete 4/7)
M00895 Thiamine biosynthesis, prokaryotes, AIR (+ DXP/glycine) => TMP/TPP (7) (incomplete 6/9)	M00895 Thiamine biosynthesis, prokaryotes, AIR (+ DXP/glycine) => TMP/TPP (7) (incomplete 6/9)	M00895 Thiamine biosynthesis, prokaryotes, AIR (+ DXP/glycine) => TMP/TPP (7) (incomplete 6/9)
M00896 Thiamine biosynthesis, archaea, AIR (+ NAD+) => TMP/TPP (4) (1 block missing 3/4)	M00896 Thiamine biosynthesis, archaea, AIR (+ NAD+) => TMP/TPP (4) (1 block missing 3/4)	M00896 Thiamine biosynthesis, archaea, AIR (+ NAD+) => TMP/TPP (4) (1 block missing 3/4)
M00897 Thiamine biosynthesis, plants, AIR (+ NAD+) => TMP/thiamine/TPP (1) (incomplete 1/5)	M00897 Thiamine biosynthesis, plants, AIR (+ NAD+) => TMP/thiamine/TPP (1) (incomplete 1/5)	M00897 Thiamine biosynthesis, plants, AIR (+ NAD+) => TMP/thiamine/TPP (1) (incomplete 1/5)
M00899 Thiamine salvage pathway, HMP/HET => TMP (2) (1 block missing 1/2)	M00899 Thiamine salvage pathway, HMP/HET => TMP (2) (1 block missing 1/2)	M00899 Thiamine salvage pathway, HMP/HET => TMP (2) (1 block missing 1/2)
M00125 Riboflavin biosynthesis, plants and bacteria, GTP => riboflavin/FMN/FAD (5) (1 block missing 6/7)	M00125 Riboflavin biosynthesis, plants and bacteria, GTP => riboflavin/FMN/FAD (5) (1 block missing 6/7)	M00125 Riboflavin biosynthesis, plants and bacteria, GTP => riboflavin/FMN/FAD (5) (1 block missing 6/7)
M00911 Riboflavin biosynthesis, fungi, GTP => riboflavin/FMN/FAD (2) (incomplete 2/8)	M00911 Riboflavin biosynthesis, fungi, GTP => riboflavin/FMN/FAD (2) (incomplete 2/8)	M00911 Riboflavin biosynthesis, fungi, GTP => riboflavin/FMN/FAD (2) (incomplete 2/8)
M00124 Pyridoxal-P biosynthesis, erythrose-4P => pyridoxal-P (4) (2 blocks missing 4/6)	M00124 Pyridoxal-P biosynthesis, erythrose-4P => pyridoxal-P (4) (2 blocks missing 4/6)	M00124 Pyridoxal-P biosynthesis, erythrose-4P => pyridoxal-P (3) (incomplete 3/6)
M00115 NAD biosynthesis, aspartate => quinolinate => NAD (5) (complete 5/5)	M00115 NAD biosynthesis, aspartate => quinolinate => NAD (4) (1 block missing 4/5)	M00115 NAD biosynthesis, aspartate => quinolinate => NAD (5) (complete 5/5)
M00912 NAD biosynthesis, tryptophan => quinolinate => NAD (3) (incomplete 3/8)	M00912 NAD biosynthesis, tryptophan => quinolinate => NAD (2) (incomplete 2/8)	M00912 NAD biosynthesis, tryptophan => quinolinate => NAD (3) (incomplete 3/8)
M00119 Pantothenate biosynthesis, valine/L-aspartate => pantothenate (3) (2 blocks missing 3/5)	M00119 Pantothenate biosynthesis, valine/L-aspartate => pantothenate (1) (incomplete 1/5)	M00119 Pantothenate biosynthesis, valine/L-aspartate => pantothenate (1) (incomplete 1/5)
M00913 Pantothenate biosynthesis, 2-oxoisovalerate/spermine => pantothenate (2) (incomplete 2/5)	**ABSENT**	**ABSENT**

M00120 Coenzyme A biosynthesis, pantothenate => CoA (4) (complete 3/3)	M00120 Coenzyme A biosynthesis, pantothenate => CoA (4) (complete 3/3)	M00120 Coenzyme A biosynthesis, pantothenate => CoA (4) (complete 3/3)
M00914 Coenzyme A biosynthesis, archaea, 2-oxoisovalerate => 4-phosphopantoate => CoA (2) (incomplete 2/7)	M00914 Coenzyme A biosynthesis, archaea, 2-oxoisovalerate => 4-phosphopantoate => CoA (1) (incomplete 1/7)	M00914 Coenzyme A biosynthesis, archaea, 2-oxoisovalerate => 4-phosphopantoate => CoA (1) (incomplete 1/7)
M00572 Pimeloyl-ACP biosynthesis, BioC-BioH pathway, malonyl-ACP => pimeloyl-ACP (6) (complete 6/6)	M00572 Pimeloyl-ACP biosynthesis, BioC-BioH pathway, malonyl-ACP => pimeloyl-ACP (6) (complete 6/6)	M00572 Pimeloyl-ACP biosynthesis, BioC-BioH pathway, malonyl-ACP => pimeloyl-ACP (5) (1 block missing 5/6)
M00123 Biotin biosynthesis, pimeloyl-ACP/CoA => biotin (4) (complete 3/3)	M00123 Biotin biosynthesis, pimeloyl-ACP/CoA => biotin (4) (complete 3/3)	M00123 Biotin biosynthesis, pimeloyl-ACP/CoA => biotin (3) (1 block missing 2/3)
M00950 Biotin biosynthesis, BioU pathway, pimeloyl-ACP/CoA => biotin (3) (1 block missing 3/4)	M00950 Biotin biosynthesis, BioU pathway, pimeloyl-ACP/CoA => biotin (3) (1 block missing 3/4)	M00950 Biotin biosynthesis, BioU pathway, pimeloyl-ACP/CoA => biotin (2) (2 blocks missing 2/4)
M00573 Biotin biosynthesis, BioI pathway, long-chain-acyl-ACP => pimeloyl-ACP => biotin (3) (2 blocks missing 3/5)	M00573 Biotin biosynthesis, BioI pathway, long-chain-acyl-ACP => pimeloyl-ACP => biotin (3) (2 blocks missing 3/5)	M00573 Biotin biosynthesis, BioI pathway, long-chain-acyl-ACP => pimeloyl-ACP => biotin (2) (incomplete 2/5)
M00577 Biotin biosynthesis, BioW pathway, pimelate => pimeloyl-CoA => biotin (4) (1 block missing 4/5)	M00577 Biotin biosynthesis, BioW pathway, pimelate => pimeloyl-CoA => biotin (4) (1 block missing 4/5)	M00577 Biotin biosynthesis, BioW pathway, pimelate => pimeloyl-CoA => biotin (3) (2 blocks missing 3/5)
M00881 Lipoic acid biosynthesis, plants and bacteria, octanoyl-ACP => dihydrolipoyl-E2/H (2) (complete 2/2)	M00881 Lipoic acid biosynthesis, plants and bacteria, octanoyl-ACP => dihydrolipoyl-E2/H (2) (complete 2/2)	M00881 Lipoic acid biosynthesis, plants and bacteria, octanoyl-ACP => dihydrolipoyl-E2/H (2) (complete 2/2)
M00882 Lipoic acid biosynthesis, eukaryotes, octanoyl-ACP => dihydrolipoyl-H (1) (1 block missing 1/2)	M00882 Lipoic acid biosynthesis, eukaryotes, octanoyl-ACP => dihydrolipoyl-H (1) (1 block missing 1/2)	M00882 Lipoic acid biosynthesis, eukaryotes, octanoyl-ACP => dihydrolipoyl-H (1) (1 block missing 1/2)
M00883 Lipoic acid biosynthesis, animals and bacteria, octanoyl-ACP => dihydrolipoyl-H => dihydrolipoyl-E2 (1) (2 blocks missing 1/3)	M00883 Lipoic acid biosynthesis, animals and bacteria, octanoyl-ACP => dihydrolipoyl-H => dihydrolipoyl-E2 (1) (2 blocks missing 1/3)	M00883 Lipoic acid biosynthesis, animals and bacteria, octanoyl-ACP => dihydrolipoyl-H => dihydrolipoyl-E2 (1) (2 blocks missing 1/3)
M00884 Lipoic acid biosynthesis, octanoyl-CoA => dihydrolipoyl-E2 (1) (1 block missing 1/2)	M00884 Lipoic acid biosynthesis, octanoyl-CoA => dihydrolipoyl-E2 (1) (1 block missing 1/2)	M00884 Lipoic acid biosynthesis, octanoyl-CoA => dihydrolipoyl-E2 (1) (1 block missing 1/2)
M00126 Tetrahydrofolate biosynthesis, GTP => THF (4) (2 blocks missing 3/5)	M00126 Tetrahydrofolate biosynthesis, GTP => THF (4) (2 blocks missing 3/5)	M00126 Tetrahydrofolate biosynthesis, GTP => THF (4) (2 blocks missing 3/5)
M00840 Tetrahydrofolate biosynthesis, mediated by ribA and trpF, GTP => THF (1) (incomplete 1/6)	M00840 Tetrahydrofolate biosynthesis, mediated by ribA and trpF, GTP => THF (1) (incomplete 1/6)	M00840 Tetrahydrofolate biosynthesis, mediated by ribA and trpF, GTP => THF (1) (incomplete 1/6)
M00841 Tetrahydrofolate biosynthesis, mediated by PTPS, GTP => THF (2) (incomplete 2/5)	M00841 Tetrahydrofolate biosynthesis, mediated by PTPS, GTP => THF (2) (incomplete 2/5)	M00841 Tetrahydrofolate biosynthesis, mediated by PTPS, GTP => THF (2) (incomplete 2/5)
M00842 Tetrahydrobiopterin biosynthesis, GTP => BH4 (2) (1 block missing 2/3)	M00842 Tetrahydrobiopterin biosynthesis, GTP => BH4 (2) (1 block missing 2/3)	M00842 Tetrahydrobiopterin biosynthesis, GTP => BH4 (2) (1 block missing 2/3)
M00843 L-threo-Tetrahydrobiopterin biosynthesis, GTP => L-threo-BH4 (2) (1 block missing 2/3)	M00843 L-threo-Tetrahydrobiopterin biosynthesis, GTP => L-threo-BH4 (2) (1 block missing 2/3)	M00843 L-threo-Tetrahydrobiopterin biosynthesis, GTP => L-threo-BH4 (2) (1 block missing 2/3)
M00880 Molybdenum cofactor biosynthesis, GTP => molybdenum cofactor (4) (1 block missing 2/3)	**ABSENT**	**ABSENT**
M00140 C1-unit interconversion, prokaryotes (2) (1 block missing 2/3)	M00140 C1-unit interconversion, prokaryotes (2) (1 block missing 2/3)	M00140 C1-unit interconversion, prokaryotes (2) (1 block missing 2/3)
M00141 C1-unit interconversion, eukaryotes (1) (1 block missing 1/2)	M00141 C1-unit interconversion, eukaryotes (1) (1 block missing 1/2)	M00141 C1-unit interconversion, eukaryotes (1) (1 block missing 1/2)
M00846 Siroheme biosynthesis, glutamyl-tRNA => siroheme (4) (2 blocks missing 4/6)	M00846 Siroheme biosynthesis, glutamyl-tRNA => siroheme (4) (2 blocks missing 4/6)	M00846 Siroheme biosynthesis, glutamyl-tRNA => siroheme (4) (2 blocks missing 4/6)
M00868 Heme biosynthesis, animals and fungi, glycine => heme (7) (1 block missing 7/8)	M00868 Heme biosynthesis, animals and fungi, glycine => heme (7) (1 block missing 7/8)	M00868 Heme biosynthesis, animals and fungi, glycine => heme (7) (1 block missing 7/8)

M00121 Heme biosynthesis, plants and bacteria, glutamate => heme (8) (2 blocks missing 8/10)	M00121 Heme biosynthesis, plants and bacteria, glutamate => heme (8) (2 blocks missing 8/10)	M00121 Heme biosynthesis, plants and bacteria, glutamate => heme (8) (2 blocks missing 8/10)
M00926 Heme biosynthesis, bacteria, glutamyl-tRNA => coproporphyrin III => heme (5) (incomplete 5/9)	M00926 Heme biosynthesis, bacteria, glutamyl-tRNA => coproporphyrin III => heme (5) (incomplete 5/9)	M00926 Heme biosynthesis, bacteria, glutamyl-tRNA => coproporphyrin III => heme (5) (incomplete 5/9)
M00924 Cobalamin biosynthesis, anaerobic, uroporphyrinogen III => sirohydrochlorin => cobyrinate a,c-diamide (9) (2 blocks missing 9/11)	M00924 Cobalamin biosynthesis, anaerobic, uroporphyrinogen III => sirohydrochlorin => cobyrinate a,c-diamide (9) (2 blocks missing 9/11)	M00924 Cobalamin biosynthesis, anaerobic, uroporphyrinogen III => sirohydrochlorin => cobyrinate a,c-diamide (6) (incomplete 6/11)
M00925 Cobalamin biosynthesis, aerobic, uroporphyrinogen III => precorrin 2 => cobyrinate a,c-diamide (11) (2 blocks missing 9/11)	M00925 Cobalamin biosynthesis, aerobic, uroporphyrinogen III => precorrin 2 => cobyrinate a,c-diamide (11) (2 blocks missing 9/11)	M00925 Cobalamin biosynthesis, aerobic, uroporphyrinogen III => precorrin 2 => cobyrinate a,c-diamide (8) (incomplete 6/11)
M00122 Cobalamin biosynthesis, cobyrinate a,c-diamide => cobalamin (7) (2 blocks missing 5/7)	M00122 Cobalamin biosynthesis, cobyrinate a,c-diamide => cobalamin (8) (complete 7/7)	M00122 Cobalamin biosynthesis, cobyrinate a,c-diamide => cobalamin (3) (incomplete 3/7)
M00117 Ubiquinone biosynthesis, prokaryotes, chorismate (+ polyprenyl-PP) => ubiquinol (4) (incomplete 5/9)	M00117 Ubiquinone biosynthesis, prokaryotes, chorismate (+ polyprenyl-PP) => ubiquinol (4) (incomplete 5/9)	M00117 Ubiquinone biosynthesis, prokaryotes, chorismate (+ polyprenyl-PP) => ubiquinol (4) (incomplete 5/9)
M00116 Menaquinone biosynthesis, chorismate (+ polyprenyl-PP) => menaquinol (1) (incomplete 1/9)	M00116 Menaquinone biosynthesis, chorismate (+ polyprenyl-PP) => menaquinol (1) (incomplete 1/9)	M00116 Menaquinone biosynthesis, chorismate (+ polyprenyl-PP) => menaquinol (1) (incomplete 1/9)
**Biosynthesis of terpenoids and polyketides**	**Biosynthesis of terpenoids and polyketides**	**Biosynthesis of terpenoids and polyketides**
Terpenoid backbone biosynthesis	Terpenoid backbone biosynthesis	Terpenoid backbone biosynthesis
M00096 C5 isoprenoid biosynthesis, non-mevalonate pathway (6) (1 block missing 7/8)	M00096 C5 isoprenoid biosynthesis, non-mevalonate pathway (6) (1 block missing 7/8)	M00096 C5 isoprenoid biosynthesis, non-mevalonate pathway (6) (1 block missing 7/8)
M00364 C10-C20 isoprenoid biosynthesis, bacteria (1) (1 block missing 1/2)	M00364 C10-C20 isoprenoid biosynthesis, bacteria (1) (1 block missing 1/2)	M00364 C10-C20 isoprenoid biosynthesis, bacteria (1) (1 block missing 1/2)
Polyketide sugar unit biosynthesis	Polyketide sugar unit biosynthesis	Polyketide sugar unit biosynthesis
M00793 dTDP-L-rhamnose biosynthesis (4) (complete 3/3)	M00793 dTDP-L-rhamnose biosynthesis (4) (complete 3/3)	M00793 dTDP-L-rhamnose biosynthesis (4) (complete 3/3)
**Biosynthesis of other secondary metabolites**	**Biosynthesis of other secondary metabolites**	**Biosynthesis of other secondary metabolites**
Biosynthesis of phytochemical compounds	Biosynthesis of phytochemical compounds	Biosynthesis of phytochemical compounds
M00953 Mugineic acid biosynthesis, methionine => 3-epihydroxymugineic acid (1) (incomplete 1/5)	M00953 Mugineic acid biosynthesis, methionine => 3-epihydroxymugineic acid (1) (incomplete 1/5)	M00953 Mugineic acid biosynthesis, methionine => 3-epihydroxymugineic acid (1) (incomplete 1/5)
**Xenobiotics biodegradation**	**Xenobiotics biodegradation**	**Xenobiotics biodegradation**
Aromatics degradation	Aromatics degradation	Aromatics degradation
M00569 Catechol meta-cleavage, catechol => acetyl-CoA / 4-methylcatechol => propanoyl-CoA (1) (incomplete 1/5)	**ABSENT**	**ABSENT**
M00878 Phenylacetate degradation, phenylaxetate => acetyl-CoA/succinyl-CoA (1) (incomplete 1/7)	M00878 Phenylacetate degradation, phenylaxetate => acetyl-CoA/succinyl-CoA (1) (incomplete 1/7)	M00878 Phenylacetate degradation, phenylaxetate => acetyl-CoA/succinyl-CoA (1) (incomplete 1/7)
M00545 Trans-cinnamate degradation, trans-cinnamate => acetyl-CoA (1) (incomplete 1/6)	**ABSENT**	**ABSENT**
**Gene set**	**Gene set**	**Gene set**
Pathogenicity	Pathogenicity	Pathogenicity

M00575 Pertussis pathogenicity signature, T1SS (3) (2 blocks missing 3/5)	M00575 Pertussis pathogenicity signature, T1SS (3) (2 blocks missing 3/5)	M00575 Pertussis pathogenicity signature, T1SS (1) (incomplete 1/5)
Drug resistance	Drug resistance	Drug resistance
M00627 beta-Lactam resistance, Bla system (1) (2 blocks missing 1/3)	M00627 beta-Lactam resistance, Bla system (1) (2 blocks missing 1/3)	**ABSENT**
M00726 Cationic antimicrobial peptide (CAMP) resistance, lysyl-phosphatidylglycerol (L-PG) synthase MprF (1) (2 blocks missing 1/3)	M00726 Cationic antimicrobial peptide (CAMP) resistance, lysyl-phosphatidylglycerol (L-PG) synthase MprF (1) (2 blocks missing 1/3)	M00726 Cationic antimicrobial peptide (CAMP) resistance, lysyl-phosphatidylglycerol (L-PG) synthase MprF (1) (2 blocks missing 1/3)
M00718 Multidrug resistance, efflux pump MexAB-OprM (3) (1 block missing 1/2)	M00718 Multidrug resistance, efflux pump MexAB-OprM (3) (1 block missing 1/2)	**ABSENT**
M00642 Multidrug resistance, efflux pump MexJK-OprM (2) (1 block missing 1/2)	**ABSENT**	**ABSENT**
**Module set**		
Metabolic capacity	**ABSENT**	**ABSENT**
M00618 Acetogen (0) (1 block missing 1/2)		

Legend:

pathway present in Asaia bogorensis genom and absent in *Ca.* Kirkpatrella diaphorinas and *Acetobacteraceae*_TRYOCC genomes

pathway present in Asaia bogorensis and *Ca.* Kirkpatrella diaphorinas genomes but absent in *Acetobacteraceae*_TRYOCC genome

pathway with missing gne(s) in *Acetobacteraceae*_TRYOCC relative to *Ca.* Kirkpatrella diaphorinas

Table S2. The comparison of gene set between *Sulcia*-TRYOCC and *Sulcia* lineages from eight planthopper species.

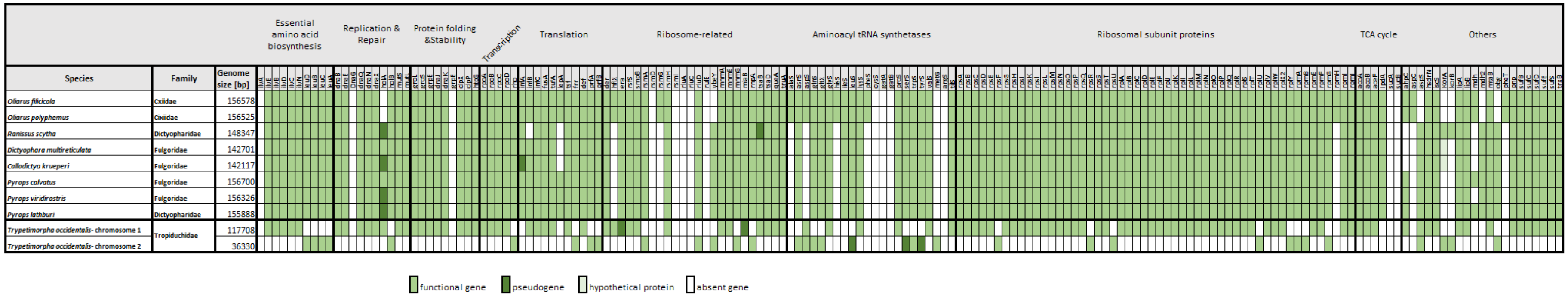

Table S3. The comparison of gene set between *Vidania*-TRYOCC and *Vidania* lineages from eight planthopper species.

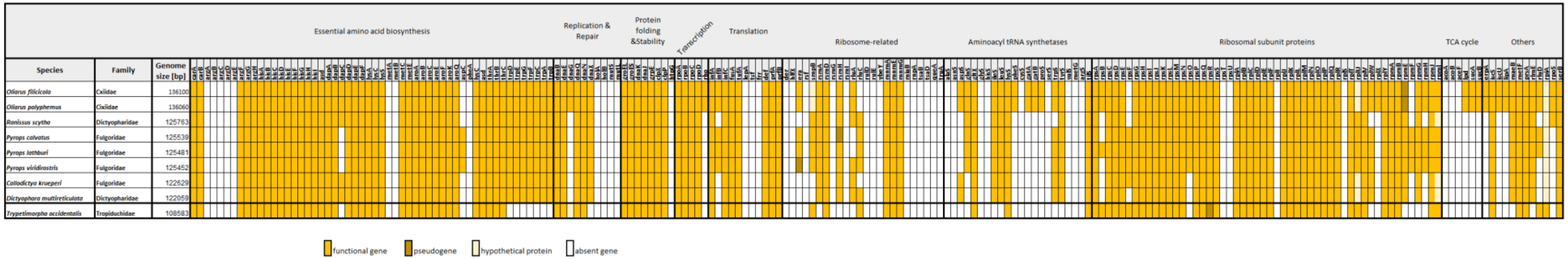

Table S4. Completeness values for *Sulcia*, *Vidania* and *Acetobacteraceae* symbionts of *T. occidentalis* and other *Sulcia* symbionts of Auchenorrhyncha.

Host	Symbiont	Genome assembly acession number	Completeness-general	Contamination	Completeness-specific	Completeness-model used	Translation table used	Coding density	Contig_N50	Average gene length	Size	GC content	Total coding sequences
Trypetimorpha occidentalis	***Acetobacteraceae***	**CP154827**	**99,41**	**0,03**	**99,9**	**Neural Network (Specific Model)**	**11**	**0,857**	**1966088**	**245,0954033**	**1966088**	**0,57**	**2306**
Kosemia yezoensis	*Sulcia*	GCF_003213475.1	97,55	0,32	89,35	Neural Network (Specific Model)	4	0,899	244201	296,8709677	244201	0,23	248
Auritibicen bihamatus	*Sulcia*	GCF_003213565.1	95,45	0,26	91,5	Neural Network (Specific Model)	4	0,931	276765	335,540856	276765	0,23	257
Meimuna kuroiwae	*Sulcia*	GCF_003214015.1	95,27	0,24	90,29	Neural Network (Specific Model)	4	0,939	271073	334,3137255	271073	0,23	255
Auritibicen japonicus	*Sulcia*	GCF_003213655.1	95,23	0,33	92,25	Neural Network (Specific Model)	4	0,932	278175	328,780303	278175	0,23	264
Vagitanus terminalis	*Sulcia*	GCF_003213775.1	95,18	0,21	93,77	Neural Network (Specific Model)	4	0,936	245295	328,5982906	245295	0,23	234
Mogannia minuta	*Sulcia*	GCF_003214365.1	94,6	0,53	94,24	Neural Network (Specific Model)	4	0,923	243548	328,6593886	243548	0,22	229
Meimuna oshimensis	*Sulcia*	GCF_003214135.1	94,36	0,23	91,33	Neural Network (Specific Model)	4	0,942	270602	332,1128405	270602	0,23	257
Meimuna iwasakii	*Sulcia*	GCF_003215395.1	94,36	0,23	91,35	Neural Network (Specific Model)	4	0,94	272319	333,4902724	272319	0,23	257
Terpnosia vacua	*Sulcia*	GCF_003213895.1	93,81	0,23	92,3	Neural Network (Specific Model)	4	0,94	273800	340,5533597	273800	0,23	253
Euterpnosia chibensis	*Sulcia*	GCF_003214255.1	93,81	0,24	92,06	Neural Network (Specific Model)	4	0,943	273706	338,7960784	273706	0,23	255
Terpnosia nigricosta	*Sulcia*	GCF_003214655.1	93,81	0,23	92,3	Neural Network (Specific Model)	4	0,939	273631	337,5568627	273631	0,23	255
Hyalessa maculaticollis	*Sulcia*	GCF_003215155.1	93,63	0,25	88,74	Neural Network (Specific Model)	4	0,927	272577	321,3612167	272577	0,22	263
Tanna japonensis	*Sulcia*	GCF_003215265.1	93,45	0,22	90,77	Neural Network (Specific Model)	4	0,926	278298	337,1054688	278298	0,22	256
Muda kuroiwae	*Sulcia*	GCF_003214495.1	93,33	0,2	92,61	Neural Network (Specific Model)	4	0,947	266952	330,6210938	266952	0,23	256
Cryptotympana atrata	*Sulcia*	GCF_003214785.1	93,29	0,16	91,38	Neural Network (Specific Model)	4	0,936	273226	333,1284047	273226	0,23	257
Graptopsaltria nigrofuscata	*Sulcia*	GCF_003213395.1	93,26	0,22	89,78	Neural Network (Specific Model)	4	0,941	271571	334,1015625	271571	0,23	256
Graptopsaltria bimaculata	*Sulcia*	GCF_003215035.1	93,26	0,22	89,78	Neural Network (Specific Model)	4	0,943	271622	334,8203125	271622	0,23	256
Meimuna opalifera	*Sulcia*	GCF_003215515.1	92,74	0,23	91,17	Neural Network (Specific Model)	4	0,939	271556	336,2480315	271556	0,23	254
Oliarus polyphemus	*Sulcia*	GCF_028673255.1	92,36	0,19	80,58	Neural Network (Specific Model)	4	0,908	156525	307,2387097	156525	0,25	155
Oliarus filicicola	*Sulcia*	GCF_003391295.1	91,7	0,18	81,83	Neural Network (Specific Model)	4	0,901	156578	306,961039	156578	0,25	154
Cryptotympana facialis	*Sulcia*	GCF_003214895.1	91,25	0,94	91,82	Neural Network (Specific Model)	4	0,934	270781	321,9695817	270781	0,23	263
Oliarus filicicola	*Sulcia*	GCF_028748865.1	90,11	0,18	71,74	Neural Network (Specific Model)	4	0,901	155965	311,8874172	155965	0,25	151
Dictyophara multireticulata	*Sulcia*	GCF_016888765.1	88,92	0,22	65,14	Neural Network (Specific Model)	4	0,921	142701	305,5902778	142701	0,26	144
Pyrops lathburii	*Sulcia*	GCF_022985515.1	87,23	0,22	71,87	Neural Network (Specific Model)	4	0,869	155888	310,9109589	155888	0,25	146
Pyrops viridirostris	*Sulcia*	GCF_022985615.1	87,15	0,23	71,16	Neural Network (Specific Model)	4	0,867	156326	300,5960265	156326	0,25	151
Macrosteles quadripunctulatus	*Sulcia*	GCF_001447915.1	86,18	0,28	82,48	Neural Network (Specific Model)	4	0,949	190657	317,9109948	190657	0,24	191
Macrosteles sp. nr. severini	*Sulcia*	GCF_014211855.1	86,18	0,28	82,48	Neural Network (Specific Model)	4	0,947	190827	319,1736842	190827	0,24	190
Ranissus scytha	*Sulcia*	GCF_016888785.1	85,79	0,21	65,77	Neural Network (Specific Model)	4	0,886	148347	305,6736111	148347	0,25	144
Entylia carinata	*Sulcia*	GCF_002243425.1	85,46	0,29	89,75	Neural Network (Specific Model)	4	0,907	218034	324,622549	218034	0,23	204
Pyrops clavatus	*Sulcia*	GCF_022985555.1	85,16	0,22	71,34	Neural Network (Specific Model)	4	0,882	156700	310,6107383	156700	0,25	149
Callodictya krueperi	*Sulcia*	GCF_016889585.1	84,71	0,23	64,52	Neural Network (Specific Model)	4	0,921	142117	304,6736111	142117	0,26	144
Platypleura kaempferi	*Sulcia*	GCF_003214575.1	84,3	0,15	88,12	Neural Network (Specific Model)	4	0,936	268037	336,28	268037	0,23	250
Trypetimorpha occidentalis	***Sulcia***	**GCA_033550855.1**	**83,83**	**0,19**	**64,76**	**Neural Network (Specific Model)**	**4**	**0,861**	**117708**	**283,3121019**	**154038**	**0,24**	**157**
Nephotettix cincticeps	*Sulcia*	GCF_001677255.1	80,79	0,2	80,21	Neural Network (Specific Model)	4	0,927	192244	300,3417085	192244	0,24	199
Nesophrosyne sp. 246	*Sulcia*	GCF_024817055.1	80,19	0,28	81,77	Neural Network (Specific Model)	4	0,93	190377	314,4232804	190377	0,24	189
Nesophrosyne sp. 21	*Sulcia*	GCF_024817195.1	80,07	0,65	82,59	Neural Network (Specific Model)	4	0,93	190814	310,25	190814	0,24	192
Nesophrosyne makaihe	*Sulcia*	GCF_024817015.1	79,96	0,28	81,51	Neural Network (Specific Model)	4	0,93	190656	316,6648936	190656	0,24	188
Nesophrosyne sp. 15	*Sulcia*	GCF_024817115.1	79,88	0,28	83,14	Neural Network (Specific Model)	4	0,937	189955	314,6526316	189955	0,24	190
Nesophrosyne maratima	*Sulcia*	GCF_024817075.1	79,74	0,26	79,42	Neural Network (Specific Model)	4	0,931	190512	316,5425532	190512	0,24	188
Nephotettix cincticeps	*Sulcia*	GCF_030297035.1	79,68	0,2	81,95	Neural Network (Specific Model)	4	0,931	192344	315,9578947	192344	0,24	190
Cicadellidae	*Sulcia*	GCF_017086585.1	79,65	0,19	77,49	Neural Network (Specific Model)	4	0,923	192240	291,8529412	192240	0,24	204
Nesophrosyne sp. 302	*Sulcia*	GCF_024817975.1	79,49	0,28	81,95	Neural Network (Specific Model)	4	0,931	190964	322,5081081	190964	0,24	185
Nesophrosyne sp. 58	*Sulcia*	GCF_024817175.1	79,42	0,28	81,07	Neural Network (Specific Model)	4	0,931	190780	322,2432432	190780	0,24	185
Nesophrosyne sp. 126	*Sulcia*	GCF_024817235.1	79,42	0,28	82,12	Neural Network (Specific Model)	4	0,932	190314	318,3101604	190314	0,24	187
Nesophrosyne sp. 17	*Sulcia*	GCF_024817215.1	79,27	0,27	80,02	Neural Network (Specific Model)	4	0,94	192793	316,9427083	192793	0,24	192
Nesophrosyne ogradyi	*Sulcia*	GCF_024817995.1	79,01	0,28	81,82	Neural Network (Specific Model)	4	0,932	190661	318,9465241	190661	0,24	187
Nesophrosyne sp. 281	*Sulcia*	GCF_024817955.1	78,97	0,28	81,79	Neural Network (Specific Model)	4	0,93	190560	318,2459893	190560	0,24	187
Nesophrosyne sp. 242	*Sulcia*	GCF_024817155.1	78,93	0,28	81,99	Neural Network (Specific Model)	4	0,934	190404	314,2526316	190404	0,24	190
Nesophrosyne sp. 295	*Sulcia*	GCF_024818015.1	78,93	0,28	81,98	Neural Network (Specific Model)	4	0,932	190867	317,3351064	190867	0,24	188
Nesophrosyne haleakala	*Sulcia*	GCF_024817655.1	78,82	0,28	84,2	Neural Network (Specific Model)	4	0,926	190781	316,9465241	190781	0,24	187
Nesophrosyne kanawao	*Sulcia*	GCF_024817095.1	78,77	0,28	83,05	Neural Network (Specific Model)	4	0,931	190852	317,2659574	190852	0,24	188
Nesophrosyne sp. 29	*Sulcia*	GCF_024817335.1	78,77	0,28	83,06	Neural Network (Specific Model)	4	0,941	189814	312,2239583	189814	0,24	192
Nesophrosyne sp. 23	*Sulcia*	GCF_024817475.1	78,2	0,63	83,12	Neural Network (Specific Model)	4	0,937	190701	312,4375	190701	0,24	192
Nesophrosyne ponapona	*Sulcia*	GCF_024817035.1	76,15	0,28	80,53	Neural Network (Specific Model)	4	0,942	192889	322,4074074	192889	0,24	189
Nesophrosyne sp. 48	*Sulcia*	GCF_024817135.1	76,15	0,28	80,53	Neural Network (Specific Model)	4	0,942	192854	323,962766	192854	0,24	188
Trypetimorpha occidentalis	***Vidania***	**CP154828**	**64,06**	**0,09**	**55,66**	**Gradient Boost (General Model)**	**11**	**0,875**	**108583**	**259,8292683**	**108583**	**0,17**	**123**

Table S5. Sequences of symbiont-specific fluorochrome-labeled oligonucleotide probes.

**Fluorescence *in situ* hybridization (FISH)**					
**Probe name**	**Probe sequence**	**Specificity**	**Fluorophore**	**Hybridization buffer**	**References**
Sul664R	CCMCACATTCCAGYTACTCC	16S rRNA gene of *Sulcia*	Cy3	1M Tris-HCl (pH 8.0) – 1 ml, 5M NaCl – 9 ml, 20% SDS – 20 µl, 30% formamide – 15 ml, water – up to 50 ml	Koga et al., 2013
Bet940R	TTAATCCACATCATCCACCG	16S rRNA gene of *Vidania*	AlexaFluor 488		Demanèche et al. 2008
Asaia 1	ATAACATCGGGAAACTGGTGCT	16S rRNA gene of *Acetobacteraceae* symbiont	Cy5		Favia et al., 2007
Asaia 2	GAAATACCCATCTCTGGATA	16S rRNA gene of *Acetobacteraceae* symbiont	Cy5		Favia et al., 2007
Sulcia_ilvB	1654 bp fragment of *ilvB* gene generated by PCR reaction using primers: *Sulcia*_ilvB_F AGTGAGGAATTACCAGCTTACAGAGCA *Sulcia*_ilvB_R CACCCTTCTGAAGCTGAGGATAC	*ilvB* gene of *Sulcia* symbiont	Cy5		This paper

Table S6 Accession numbers of *Acetobacteraceae* genomes used for phylogenomic analysis.

**Genome**	**Bioproject**	**Biosample**	**Assembly**
Acetobacter pomorum	PRJNA397225	SAMN07682859	GCA_002456135.1
Acetobacter tropicalis DM	PRJNA397225	SAMN07452474	GCA_002549835.1
Acetobacter tropicalis	PRJNA642854	SAMN15399467	GCA_014486685.1
Acetobacter aceti	PRJNA311264	SAMN04396917	GCA_002005445.1
Gluconacetobacter johannae	PRJNA627338	SAMN14650889	GCA_014174305.1
Gluconacetobacter liquefaciens	PRJNA627338	SAMN14766534	GCA_014174285.1
Gluconacetobacter diazotrophicus	PRJNA21071	SAMN02598444	GCA_000021325.1
Asaia platycodi	PRJDB613	SAMD00002827	GCA_000614545.1
Asaia prunellae	PRJDB589	SAMD00004065	GCA_000613885.1
Asaia siamensis	PRJDB10511	SAMD00244847	GCA_014635085.1
Asaia astilbis	PRJDB587	SAMD00009367	GCA_000613845.1
Asaia bogorensis OTPB	PRJDB519	SAMD00019081	GCA_001547995.1
Asaia sp. SF2	PRJNA219210	SAMN02469759	GCA_000505765.1
Asaia lannensis	PRJNA849918	SAMN29145435	GCA_024054035.1
Asaia bogorensis MRS	PRJNA756835	SAMN20927460	GCA_019823045.1
Asaia bogorensis W19	PRJNA427835	SAMN08274829	GCA_003994335.1
Ca. Kirkpatrickella diaphorinae	PRJNA880923	SAMN30877677	GCA_025736875.1
Kozakia baliensis	PRJNA311264	SAMN04396915	GCF_001787355.1
Neoasaia chiangmaiensis	PRJNA311264	SAMN04396918	GCA_002005465.1
Acetobacter methanolica	PRJNA500315	SAMN10363313	GCA_004346035.1
Saccharibacter floricola	PRJNA181373	SAMN02440467	GCA_000378165.1
Saccharibacter sp. 17.LH.SD	PRJNA596238	SAMN13615857	GCA_009834805.1
Acetobacteraceae bacterium strain 880 chromosome	PRJNA532976	SAMN11430874	CP039459.1
Acetobacteraceae bacterium strain 868 chromosome	PRJNA532976	SAMN11430873	CP039460.1
Gluconobacter oxydans	PRJNA188081	SAMN05771126	GCA_000583855.1
Gluconobacter morbifer	PRJNA73361	SAMN02470912	GCA_000234355.2

References

1. Koga R, Bennett GM, Cryan JR, Moran NA. 2013. Evolutionary replacement of obligate symbionts in an ancient and diverse insect lineage. Environ Microbiol 15:2073–2081.
2. Demanèche S, Sanguin H, Poté J, Navarro E, Bernillon D, Mavingui P, Wildi W, Vogel TM, Simonet P. 2008. Antibiotic-resistant soil bacteria in transgenic plant fields. Proc Natl Acad Sci USA 105:3967–3962.
3. Favia G, Ricci I, Damiani C, et al. 2007. Bacteria of the genus *Asaia* stably associate with *Anopheles stephensi*, an Asian malarial mosquito vector. Proc Natl Acad Sci USA 104:9047–9051.
